# Sperm Nuclear Architecture Is Locally Modified in Presence of a Robertsonian Translocation t(13;17)

**DOI:** 10.1371/journal.pone.0078005

**Published:** 2013-10-31

**Authors:** Hervé Acloque, Amélie Bonnet-Garnier, Florence Mompart, Alain Pinton, Martine Yerle-Bouissou

**Affiliations:** 1 INRA, UMR444 Génétique Cellulaire, Castanet Tolosan, France; 2 Université de Toulouse, INP, ENVT, UMR444 Génétique Cellulaire, Toulouse, France; Ecole Normale Superieure de Lyon, France

## Abstract

In mammals, the non-random organization of the sperm nucleus supports an early function during embryonic development. Altering this organization may interfere with the zygote development and reduce fertility or prolificity. Thus, rare studies on sperm cells from infertile patients described an altered nuclear organization that may be a cause or a consequence of their respective pathologies. Thereby, chromosomal rearrangements and aneuploidy can be studied not only for their adverse effects on production of normal/balanced gametes at meiosis but also for their possible impact on sperm nuclear architecture and the epigenetic consequences of altered chromosome positioning. We decided to compare the global architecture of sperm nuclei from boars, either with a normal chromosome composition or with a Robertsonian translocation involving chromosomes 13 and 17. We hypothesized that the fusion between these chromosomes may change their spatial organization and we examined to what extend it could also modify the global sperm nuclear architecture. Analysis of telomeres, centromeres and gonosomes repartition does not support a global nuclear disorganization. But specific analysis of chromosomes 13 and 17 territories highlights an influence of chromosome 17 for the positioning of the fused chromosomes within the nucleus. We also observed a specific clustering of centromeres depending of the chromosome subtypes. Altogether our results showed that chromosome fusion does not significantly alter sperm nucleus architecture but suggest that centromere remodelling after chromosome fusion locally impacts chromosome positioning.

## Introduction

Recent advances on interphase nuclear imaging in its naïve 3-dimensional configuration [1] and innovative molecular tools to analyze interchromosomic interaction [2], [3] allowed deciphering the spatial organization of animal cell nuclei in relation with cellular and consequently transcriptional activity. It appears that chromosomes occupy discrete regionalized locations in the cell nucleus that corresponds to specific regions named chromosome territories (CTs) (for review [4]). This non-random organization probably relies on functional constrains to regulate genome activity, ie regulation of gene expression. Thus, the nuclear environment could favors gene specific interactions between distant genomic regions to activate or repress their transcriptional activities [5], [6] but also modulate genes position within their respective chromosome territories, depending on their expression status [7]–[10]. However to date little is known on how this three-dimensional organization is controlled and transmitted through cell division but also through generations.

One hypothesis can be that part of this information is contained in gamete nuclei before fertilization and thus will be established during spermatogenesis or oogenesis. Indeed, spermatogenesis is a complex process where the diploid genome of the spermatogonia is profoundly reprogrammed and remodeled leading to a newly highly compacted haploid genome, within the spermatozoa [11]. Shortly after meiosis, histones are going to be displaced from the chromatin and substituted by protamines, the final small basic DNA-packaging proteins [12]. Despite these complex modifications, genetic and epigenetic information is conserved and transmitted after fertilization to the zygote.

It has been shown in mammalian species that chromosomes are non-randomly localized within the sperm nucleus [13]–[16] suggesting a functional significance of such organization. But, the way chromosomes are compacted and distributed within the sperm nucleus during spermatogenesis have been to date poorly explored.

Evidences that specific epigenetic marks are transmitted through generation by the gametes are emerging [17]–[20] but the importance of chromosome localization in sperm for subsequent embryonic development after fertilization remains elusive. In animals, positioning of chromosome territories in sperm depends on species and chromosomes. Studies in chicken sperm [16], [21] and old studies in amphibians [22] support a random distribution of chromosome within the sperm nucleus while studies in mammals and monotremes globally support a non-random chromosome positioning in sperm [13]–[16], [23]–[25]. However some exceptions exist, thus chicken microchromosomes are mostly located in the central region of the sperm nucleus [16] and human chromosome 13 is found randomly distributed within the sperm nucleus [26]. It was proposed that the non-random position of X chromosome in human and marsupial sperm was linked to its propensity for inactivation [16], a process that does not exist in birds where dosage compensation by global inactivation of one sexual chromosome is absent [27]. If the function of non-random autosomes positioning in eutherian sperm nucleus remains unclear, it may be related to the dynamics of genome decompaction and genes expression just after fertilization, during the early stages of embryonic development.

Interfering with chromosome organization in sperm nucleus will help understanding how it influences gene expression at fertilization and later. Thereby, chromosomal rearrangements and aneuploidy could be studied not only for their adverse effects on production of normal/balanced gametes at meiosis but also for their possible impact on sperm nuclear architecture and the epigenetic consequences of altered chromosome positioning [28]. To date, most of the work made in sperm cells from carriers of reciprocal or Robertsonian translocation was focused on meiosis segregation analysis, and only one study looked at their effects on sperm nuclear organization in infertile patients [29]. In germ cells from heterozygous mice carriers of multiple Robertsonian translocations, Garagna et al. (2001) [30] show defects in spermatogenesis and an altered nuclear organization in spermatocytes and spermatids.

To go further with these analyses we decided to compare the global architecture of sperm nuclei from boars, either with a normal chromosome composition or with a Robertsonian translocation involving chromosomes 13 and 17 (thereafter mentioned as t(13;17)). We hypothesized that the fusion between these two chromosomes may change the spatial organization of these two chromosomes and more generally we examined to what extend it could modify the global sperm nuclear architecture, by looking at centromeres, telomeres and unaffected chromosomes (X and Y).

## Materials and Methods

### Animals

A boar heterozygous for a Robertsonian translocation t(13;17) was identified within the national systematic control program of young pedigree boars for artificial insemination centers [31], [32]. The boar semen parameters (concentration, motility and morphology) were normal and the animal was fertile [32]. Sperm form fertile boars were sampled at the Artificial Insemination Center from Rouillé (Agreement number: C8621301). This study was carried out in strict accordance of the french law regarding animal experimentation. The protocol was approved by the Committee on the Ethics of Animal Experiments of the Poitou-Charentes Province (Permit Number: CE2012-12).

### DNA Probes used in the Study

DNA oligonucleotide C6dT probes were generated from the two sequences SSCSR2A (5′-agcgcttgcctagttctcacctagc-3′, [33]) and AC6 (5′-attccatgcagcgtgattga-3′, [34]) specific of two classes of centromeric satellite sequences that are located on (sub)metacentric and acrocentric chromosomes respectively. SSCSR2A and AC6 oligonucleotides were respectively labeled with Alexa488 and Cy3 using ARES DNA labeling kit (Invitrogen) and used at a final concentration of 1 and 2 ng/µl, respectively. A biotinilated (C_3_TA_2_)_3_ LNA oligonucleotide (gift from Christophe Escudé) was used at a final concentration of 1 µM to label all the telomeres.

SpermFISH was carried out using whole chromosome painting probes for chromosomes 13 (*Sus scrofa domestica 13,* SSC13), 17 (SSC17), X (SSCX) and Y (SSCY) generated from flow sorted chromosomes [35] or commercially acquired from the University of Cambridge. The probes were amplified using DOP-PCR [36] or PARM-PCR [37] and labeled by random priming with Alexa 488 and 568 (Invitrogen).

### 3D-SpermFISH

3D-FISH experiments were carried out according to [38] with some modifications. Briefly sperm cells were washed in Phosphate Buffer Saline (PBS), spread on slide, fixed for 10 minutes with 4% paraformaldehyde in PBS, washed three times in PBT (PBS+0.5% Triton X-100), one time in 0.2 M Tris-buffer pH 8.6 and then decondensed according to Hassanane et al. (1999) [39], using a 10 mM dithiothreitol (DTT), 1.25% (w/v) papain solution in 0.2 M Tris-buffer pH 8.6 at room temperature. Optimal decondensation times were obtained between 3 and 5 min. Cells were then washed in PBS and permeabilized in PBS with 0.5% Triton X-100 for 10 minutes and washed again in PBS. Lymphocytes were isolated from freshly drawn venous blood of the control and t(13;17) animals and a dense cell suspension was applied to slides coated with poly-L-Lysine. Slides were then treated as previously described [38]. For hybridization, slides were rehydrated in 2X Saline Sodium Citrate (SSC) for 5 min and incubated in 2XSSC/50% formamide for two hours at 4°C. The probes were then dropped onto the slides and placed into a DAKO hybridizer. Cells and probes were simultaneously heat-denaturated at 72°C for 8 min and incubated overnight at 37°C for centromeric and telomeric probes and 48 h at 37°C for the chromosome painting probes. Slides were successively washed two times in 2X SSC/50% formamide for 5 min at 42°C, two times in 2X SSC for 5 min at 42°C and 2 times in 0.5X SSC for 5 min at 42°C. Slides were then mounted in Vectashield mounting media (Vector Labs) with DAPI.

### Image Acquisition and Analysis

Confocal microscopy was carried out using a Leica TCS SP2 confocal microscope equipped with an oil immersion objective (plan achromatic ×63 N.A 1.4). The Z-stacks were acquired at 1,024×1,024 pixels per frame using 8-bit pixel depth for each channel at a constant voxel size of 0.093×0.093×0.244 µm. Typically, a stack of 30 confocal planes was acquired. Segmentations and 3D measurements between objects (nucleus and CT) were done using NEMO [40], developed from Smart 3D-FISH software [41]. All objects are detected automatically by the intensity of pixels above a globally set threshold. After processing, users visually inspect image segmentation and distance values using the NEMO interface. If necessary, the segmentation threshold can be adjusted manually to improve object detection.

### Statistical Analysis

#### Centromere and telomere associations

To determine whether centromeres aggregate to form chromocenters in spermatozoa, the number of hybridization spots (AC6 and SSCSR2A probes) was determined on 3D projections obtained from confocal images using NEMO and ImageJ. A mean number of 100 nuclei were analyzed for each condition and for each probe. The same procedure was applied to the analysis of telomere associations. The resulting data consist in a series of spot numbers observed in the studied cells. We considered that the number of observations is large enough to use Gaussian asymptotic results and then a classical Student test to conclude about the existence of differences between the normal and t(13;17) animals.

#### Position of chromosome territories

To determine the position of one CT relative to the other CT, we defined three categories (colocalized, adjacent, distant). The colocalized category includes nuclei with CT that shares at least two pixels with the other segmented CT. The adjacent category comprises CTs located at less than one pixel from the edge of the other segmented CT. The distant category includes all the rest. A χ2 test was used to test against the null hypothesis that there is a random distribution between these categories. p values <0.05 were considered as significant.

Regarding relative position of CTs in the sperm nucleus we used a classical Student test to conclude about the existence of differences between relative positions of SSCX and SSCY, SSC13 and SSC17 and for each chromosome, in normal and t(13;17) animals.

## Results

### Telomere Repartition in Sperm Nuclei

**Figure 1:**
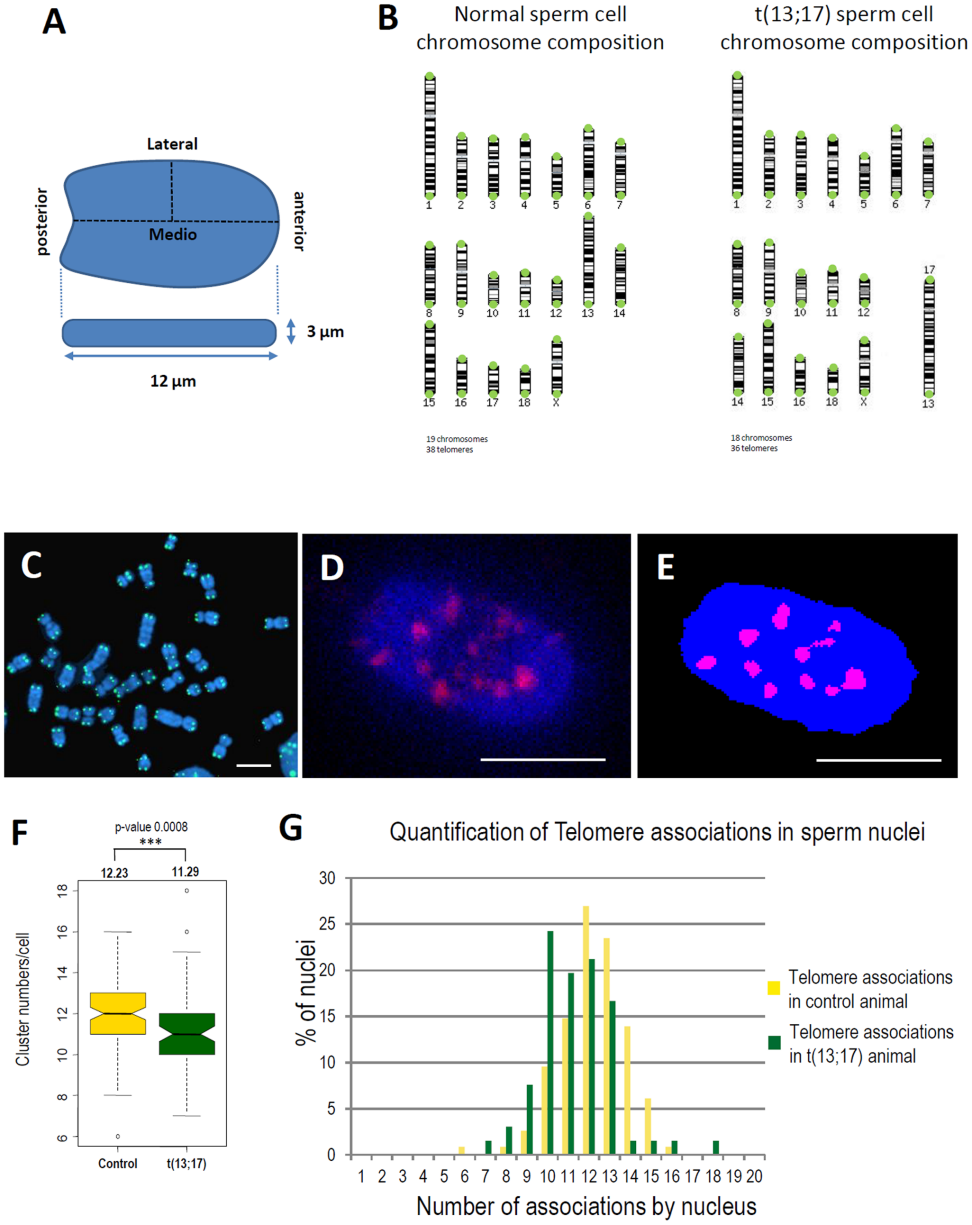
Telomere repartition in sperm nuclei. A. Morphology and dimensions of the porcine spermatozoa nucleus. B. Chromosome composition of the sperm cell from control animal with 18 autosomes and one sexual chromosome (X or Y), 38 telomeres in total and of the translocated sperm cell t(13;17) with 17 autosomes and one sexual chromosome (X or Y), 36 telomeres in total. C. DNA-FISH with telomere-specific LNA probes on porcine metaphase spreads. D–E. 3D-SpermFISH with a telomere-specific LNA probe and the resulting image segmentation obtained using NEMO. F. Boxplot representing the number of telomeres clusters in control and t(13;17) sperm nuclei. Mean values in each condition are represented above the boxplot with the p-value of the corresponding t-test. G. Observed distribution of telomere associations per nucleus in sperm nucleus of control (gold yellow) and t(13;17) (dark green) animals.

Pig sperm cells exhibit a particular morphology very similar to a paddle with a flat head (around 3 µm in depth) and a long antero-posterior axis (12 µm) and are quite different to sperm cells from human (with an ovoid shape) or mouse (with a typical hook morphology) (Fig. 1A). The nucleus contains 18 autosomes and one sexual chromosome leading to a total of 19 chromosomes and 38 telomeres (Fig. 1B).

To study the spatial repartition of telomeres in spermatozoa we used a telomere-specific LNA probe which labels all the telomeres (Fig. 1C). Telomeres are homogenously distributed within the sperm nucleus (Fig. 1D–1E). Assuming that telomeres do not cluster together, we would expect 38 specific spots corresponding to the 38 telomeres present on the 19 chromosomes. However, as shown on figures 1F–G (yellow bars), we observed a mean of 12 signals per nucleus, ranging from 6 to 16 signals per nucleus.

We then compared the telomere distribution in spermatozoa from a normal boar and from one carrier of a Robertsonian translocation t(13;17) that results from the fusion of two acrocentric chromosomes SSC13 and SSC17. We do not observed a global change of telomere repartition due to the presence of this translocation. However fewer clusters per cell were observed (mean of 11 clusters/nucleus) and this difference was significantly different from the control condition (Fig. 1F–G, green bars). The fact that the number of telomere clusters decreases was expected due to the fusion between two chromosomes in around 50% of the cells.

### Centromeres from Acrocentric Chromosome do not Cluster with Centromeres from Metacentric and Submetacentric Chromosomes

**Figure 2:**
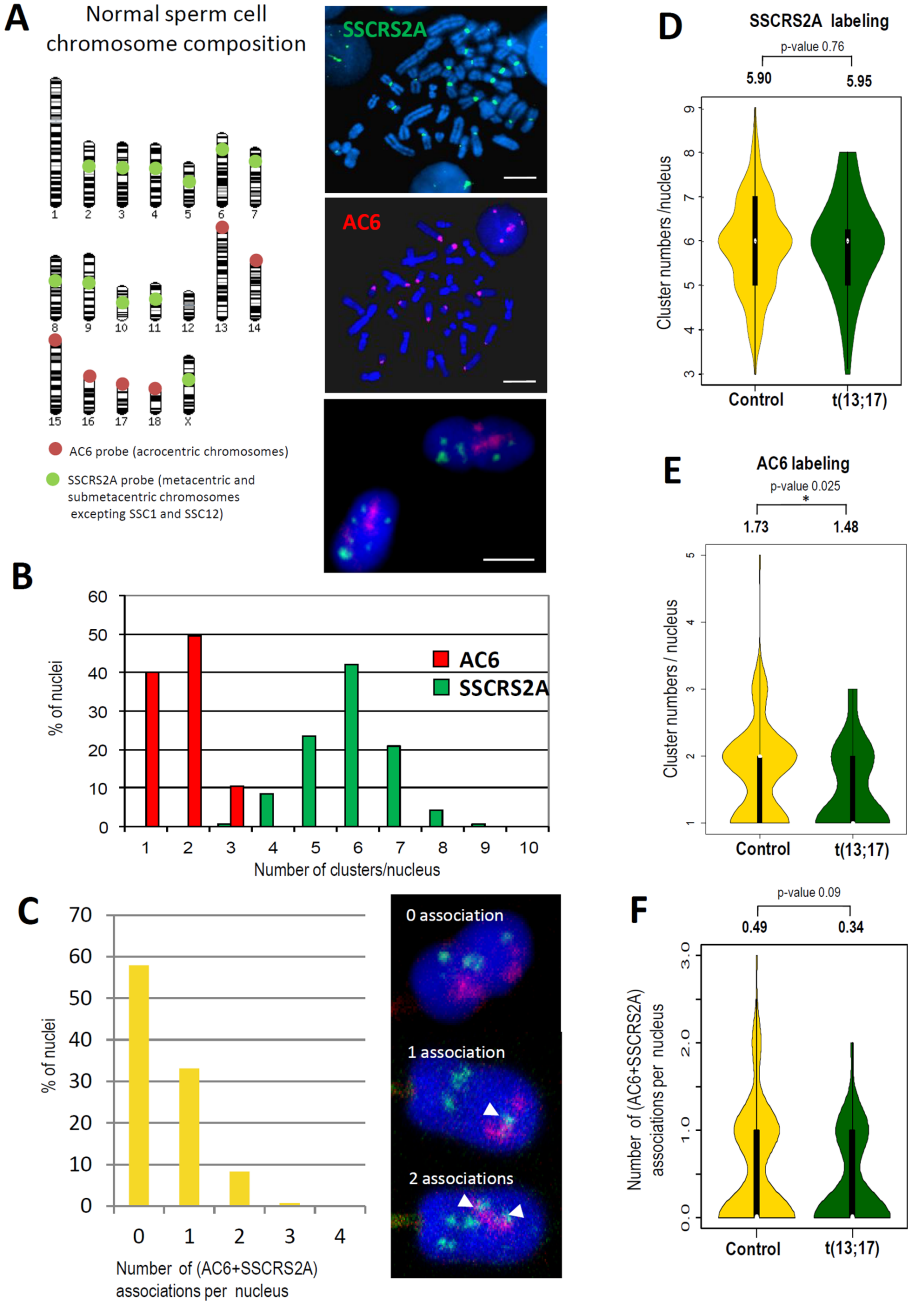
Distribution of chromocenters in sperm nuclei. A. Representation of centromeres labeled either by AC6 or SSCRS2A probes. AC6 (red dots) specifically labeled the centromere of the six porcine acrocentric chromosomes (SSC13,14,15,16,17 and 18) and SSCSR2A (green dots) specifically labeled the centromeres of the porcine chromosomes SSC2 to SSC11 and SSCX or SSCY (11 centromeres). Centromeres from SSC1 and SSC12 are not labeled by these probes. Specific labeling was checked on porcine metaphase spreads and 3D-SpermFISH was performed on sperm nucleus. B. Distribution of chromocenters in control sperm nuclei using AC6 and SSCRS2A probes. C. Percentage of nuclei containing associations of chromocenters for both AC6 and SSCR2A labeling. Arrowheads highlight associations in three different cases exemplified here: no association, 1 association or 2 associations D–E. Violin plots representing chromocenters distribution in sperm nuclei from control (gold yellow) and t(13;17) animals. Significant differences were observed for AC6 labeling (E) with less AC6 positive chromocenters in t(13;17) animal. F. Violin plots representing the number of nuclei with AC6 and SSCRS2A associations in sperm nuclei from control and t(13;17) animals.

To analyze the centromere repartition in pig sperm cells, we used two previously described centromeric probes: AC6 that recognizes α-satellite sequences specific of centromeric regions from all porcine acrocentric chromosomes (SSC13 to SSC18) and SSCRS2A that recognizes α-satellite sequences specific of centromeric regions from submetacentric and metacentric chromosomes (all other chromosomes except of SSC1 and SSC12) [37] (Fig. 2A). Centromeres from acrocentric chromosomes generally form 1 or 2 chromocenters per nucleus (representing 90% of the nuclei observed) and in some rare cases 3 chromocenters (10%) with a global mean of 1.7 chromocenters per nucleus. Each AC6 positive chromocenter contains a mean of 3.5 centromeres. Interestingly centromeres labeled with the SSCRS2A probe (most of the metacentric and submetacentric chromosomes) form much more clusters per nucleus that consequently contain less centromeres. Data analysis shows that the number of SSCRS2A positive chromocenters range from 3 to 9 per nucleus with 80% of the nuclei containing between 5 and 7 chromocenters (Fig. 2B) and a global mean of 5.9 SSCRS2A positive chromocenters per nucleus. Each SSCRS2A positive chromocenter contains a mean of 1.86 centromeres.

Moreover, we noticed very rare colocalizations of chromocenters labeled by AC6 and SSCRS2A probes. Close to 60% of the analyzed cells do not exhibit any colocalization, while 30% of the cells contain only one colocalized cluster and only 8% contain two (Fig. 2C).

We next compared the centromere repartition between spermatozoa from a normal boar and from a boar carrier of a Robertsonian translocation t(13;17). We did not observe any significant differences concerning the numbers of SSCRS2A positive chromocenters (Fig. 2D) but we observed less chromocenters labeled by AC6 probes in the sperm from the t(13;17) animal (1.73 clusters/cell against 1.48 clusters/cell) (Fig. 2E). This is coherent with the fact that an AC6 positive centromere is lost due to the centromeric fusion between SSC13 and SSC17. Regarding the number of AC6 and SSCRS2A clusters colocalization we did not observe any significant differences, even if less associations were seen in the t(13;17), with a mean of 0.34 association per nucleus against 0.49 in the control animal. Altogether these results confirm that centromeres do not cluster randomly and associate preferentially with centromeres of the same chromosome subtype, labeled either by AC6 or SSCRS2A.

### Sexual Chromosomes are Differently Localized in the Sperm Nucleus but their Respective Positions are not Affected by a Robertsonian Translocation t(13;17)

**Figure 3:**
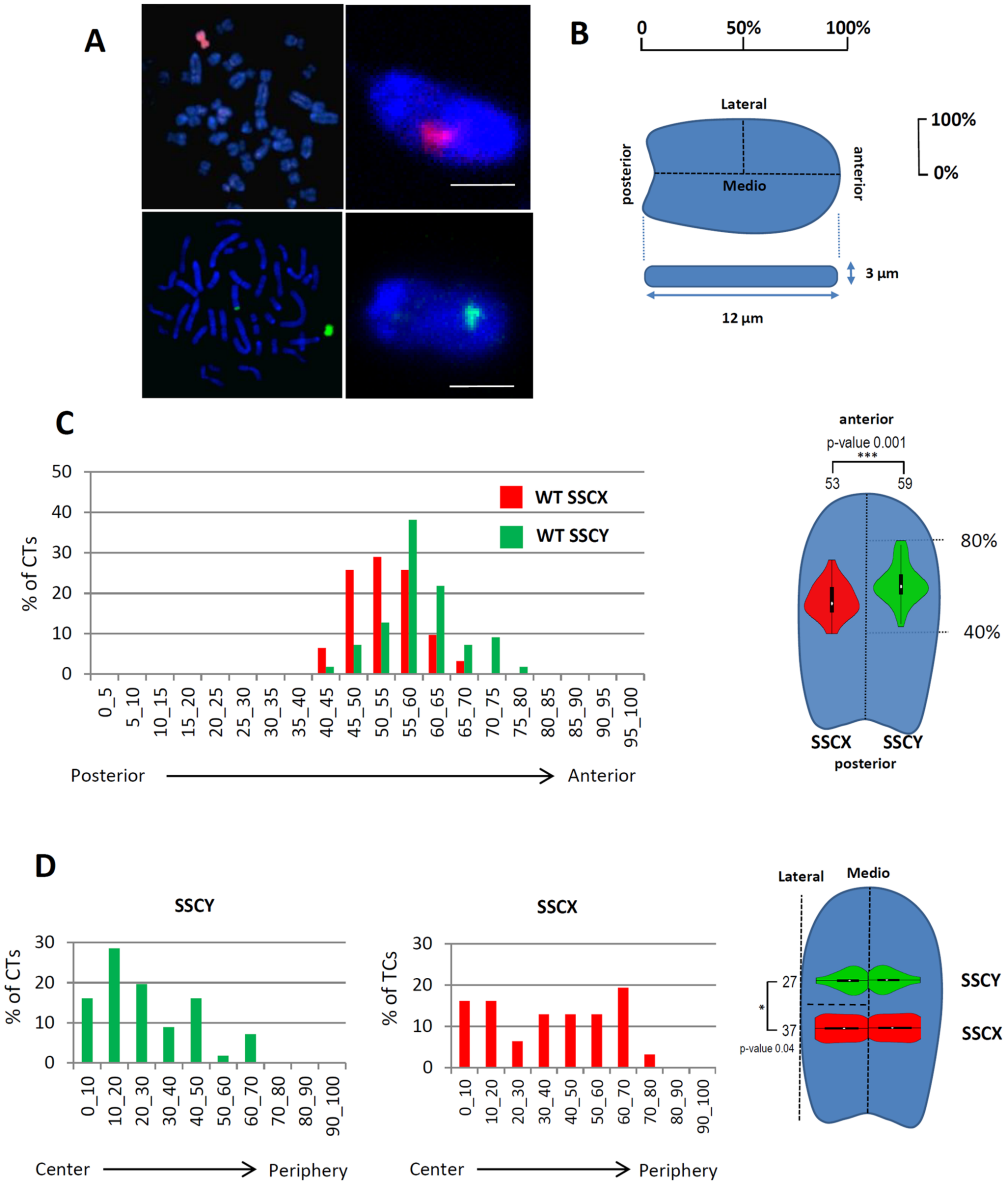
SSCX and SSCY are non random localized in the sperm nucleus. A. Chromosome paints for SSCX (red) and SSCY (green) were checked on metaphase spreads and 3D-SpermFISH was performed on sperm nuclei. B. Spatial representation of the different axis studied. Regarding the antero-posterior axis, 0% represents the most posterior part of the axis while 100% the more anterior part. For the medio-lateral axis, 0% represents the center and 100% the border of the nucleus. This axis is not lateralized and represents similarly left and right localization. C. SSCX and SSCY distributions along the anterior axis in control sperm nuclei. Note that SSCY is significantly more apical than SSCX and that the localization of sexual chromosomes is not random. SSCX and SSCY distributions are also visualized on violin plots inserted within the sperm nucleus head to visualize their respective position along the antero-posterior axis. Mean values for each chromosome are represented above the violinplot with the p-value of the corresponding t-test. D. SSCX and SSCY distributions along the medio-lateral axis in control sperm nuclei. Note that SSCX is uniformly distributed along the axis while SSCY is preferentially in the center region. SSCX and SSCY distributions are also visualized on violin plots inserted within the sperm nucleus head to visualize their respective position along the medio-lateral axis. Mean values for each chromosome are represented above the violinplot with the p-value of the corresponding t-test.

To check whether the Robertsonian translocation t(13;17) can indirectly affect the localization of other chromosomes, we looked at the position of the territories corresponding to the sex chromosomes SSCX and SSCY. Painting probes for SSCX and SSCY were validated on metaphases (Fig. 3A) and 3D-FISH was performed on sperm cells (Fig. 3A). As expected, a single sexual chromosome was observed in each cell, following mendelian inheritance ratios, both SSCX and SSCY territories exhibiting a round morphology.

We first compared SSCX and SSCY positions within the sperm nucleus. We localized the position of each CT along the antero-posterior and medio-lateral axis as described in Figure 3B. SSCX and SSCY CTs are located in the apical half of the sperm nucleus but SSCY is located more apically than SSCX (59% versus 53% along the antero-posterior axis, Fig. 3C). Similarly we observed that SSCY was localized more internally than SSCX, which is located randomly along the medio-lateral axis (Fig. 3D).

**Figure 4:**
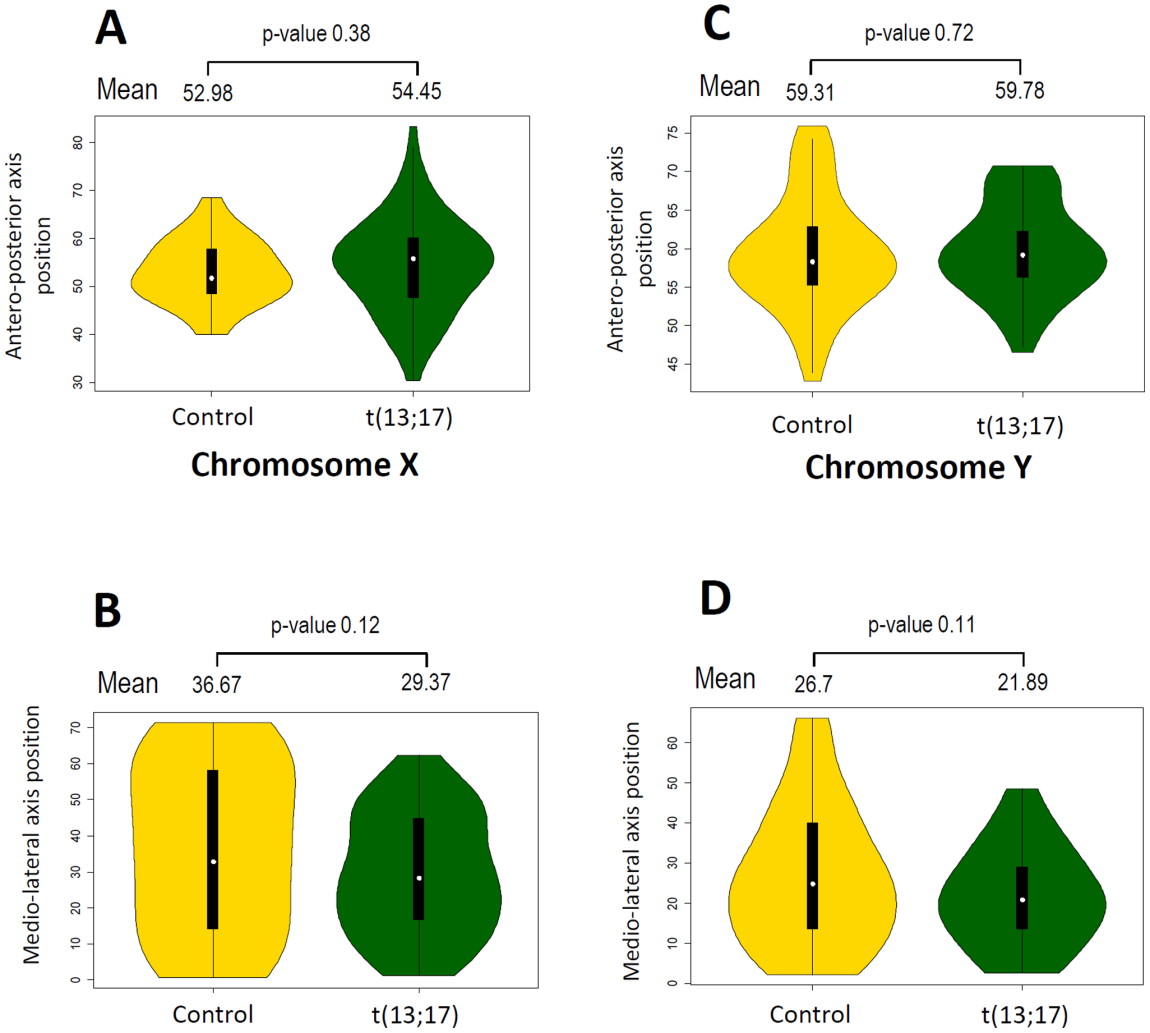
Robertsonian translocation t(13;17) does not affect SSCX and SSCY localization in the sperm nucleus. A–B. Comparison of SSCX distribution along the antero-posterior (A) and medio-lateral (B) axis in sperm nuclei from control (gold yellow) and t(13;17)(dark green) animals. Mean values for each condition are represented above the violinplot with the p-value of the corresponding t-test. C–D. Comparison of SSCY distribution along the antero-posterior (A) and medio-lateral (B) axis in sperm nuclei from control (gold yellow) and t(13;17)(dark green) animals. Mean values for each condition are represented above the violinplot with the p-value of the corresponding t-test.

We then compared the localization of SSCX and SSCY in control and t(13;17) sperm cells (Fig. 4). As for the control, SSCY was located more apically and more internally than SSCX in t(13;17) sperm cells. Regarding each specific chromosome, we observed that SSCX territory is located more apically in t(13;17) sperm cells than in the control animal (54.45% vs 52.98% along the antero-posterior axis respectively) but this difference is not significant (p-value 0.38, Fig. 4A). Regarding the medio-lateral positioning, SSCX and SSCY from t(13;17) sperm cells were located more internally (29.37% vs 36.67% and 21.89% vs 26.7% respectively, along the medio-lateral axis) but again the difference was not significant (Fig. 4B and Fig. 4D). In addition to the observations made on centromeres and telomeres clusters, our results suggest that the Robertsonian translocation t(13;17) does not significantly affect the global architecture of the sperm nucleus.

### The Robertsonian Translocation t(13;17) Affects SSC13 Localization in Sperm Cells

**Figure 5:**
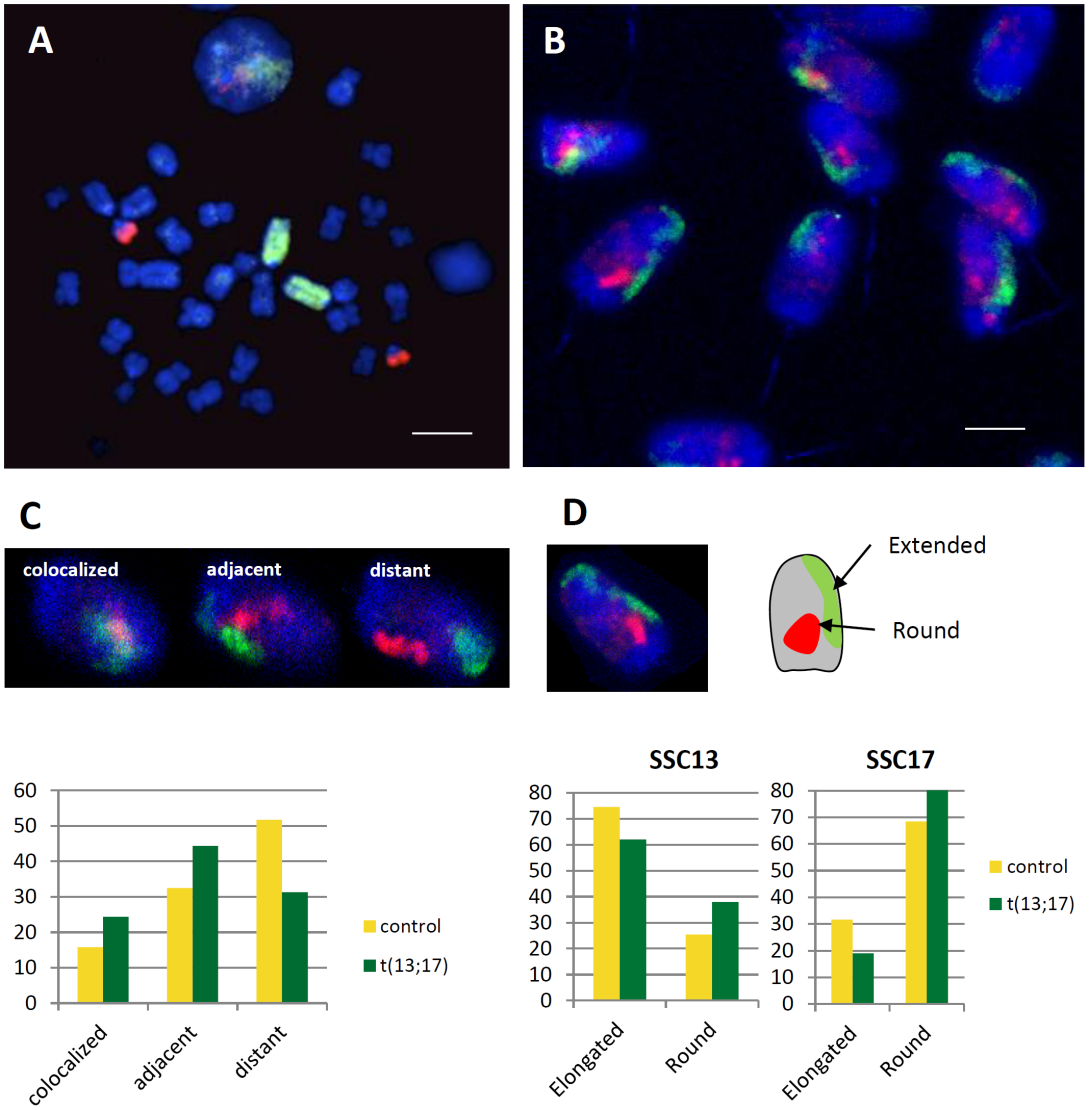
Robertsonian translocation t(13;17) affects SSC13 and SSC17 morphologies and localizations. A–B. Chromosome paints for SSC13 (green) and SSC17 (red) were checked on metaphase spreads and 3D SpermFISH was performed on sperm nuclei. C. SSC13 and SSC17 chromosome territories (CT) were classified in three categories: colocalized when territories partially or completely merged together, adjacent when they are in close contact (but not merged) and distant. Sperm nuclei were classified in these three categories and their distribution tested between control and t(13;17) animals using a χ^2^ test. D. SSC13 and SSC17 CTs were classified regarding their round or elongated morphologies. For each chromosome, sperm nuclei were classified in these two categories and their distribution tested between control and t(13;17) animals using a χ^2^ test.

Even if we did not observe significant differences in the global organization of the sperm nucleus we supposed that the fusion of chromosome 13 and 17 would affect the position of one or both chromosomes. Painting probes for SSC13 and SSC17 were validated on metaphases (Fig. 5A) and 3D-SpermFISH was performed on normal sperm and sperm from boar with a Robertsonian translocation t(13;17) (Fig. 5B). For qualitative analysis, we classified CTs localization in three different categories: colocalized, adjacent and distant (Fig. 5C) as described in Materials and Methods.

In control animal, 16% of the CTs colocalized and 32% are adjacent suggesting that these two CTs are naturally close in the sperm nucleus. This is also true in lymphocytes with 71% of the control cells nuclei that contains adjacent or colocalized SSC13 and SSC17 CTs (not shown). Performing the same analysis in t(13;17) animal, the differences were more pronounced and significantly different (p-value = 0.001) with 24% of colocalized CTs and 51% of adjacent. The t(13;17) animal being heterozygous for the translocation, half of the sperm cells carries the translocated chromosome [32]. So one half of the cells should contain only colocalized or adjacent positions while the other half is like the control condition (one half with both colocalized or adjacent positions and one half with distant positions). Taking this point in consideration, the observed proportion of CTs with colocalized or adjacent positions (75%) and distant positions (25%) in the t(13;17) sperm cells is not different from the expected ones.

A previous report by Foster et al. (2005) [15] described different morphologies between CTs in sperm nuclei. Interestingly SSC13 exhibits predominantly an elongated morphology while SSC17 is mostly round-shaped. We checked whether the fusion of these two chromosomes will affect their respective morphologies (Fig. 5D). Thus we compared the morphology of each CT in normal and t(13;17) animals. In both situation, the predominant forms were maintained (elongated for SSC13 and round for SSC17) but we observed a significant increase of the number of round CTs in sperm cells from t(13;17) animals (47% of round CTs in control animal versus 59.5% in t(13;17), p-value = 0.009 ) suggesting that the fusion affects the establishment of an elongated morphology.

**Figure 6:**
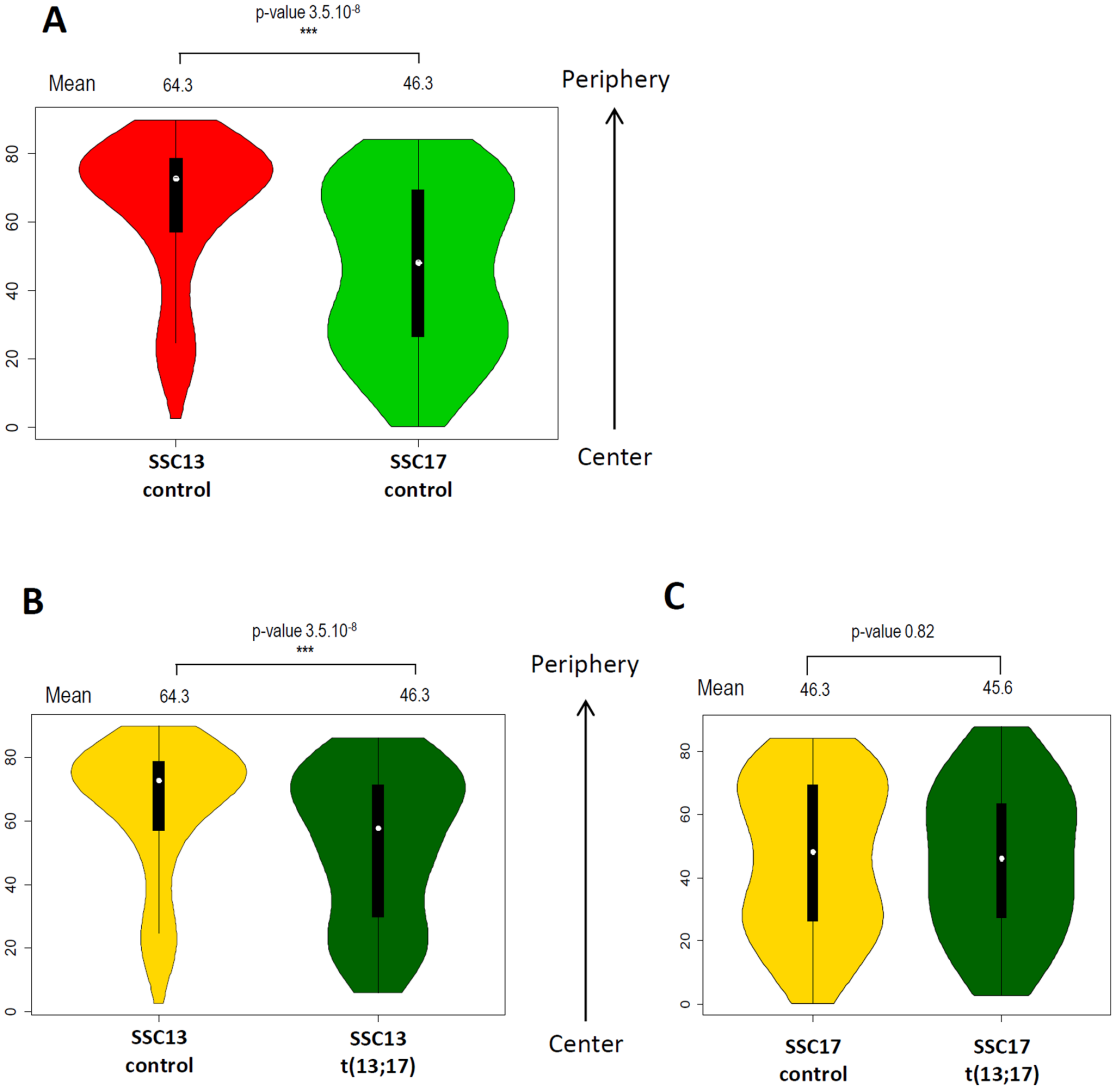
Robertsonian translocation t(13;17) affects SSC13 localization along the medio-lateral axis. A. Comparison of SSC13 and SSC17 distribution along the medio-lateral axis in control sperm nuclei. SSC13 is preferentially located at the periphery, close to the nucleus border while SSC17 is more uniformly distributed, from the periphery to the center of the nucleus. B. Comparison of SSC13 distribution along the medio-lateral axis in sperm nuclei from control (gold yellow) and t(13;17) (dark green) animals. SSC13 is displaced toward the center of the nucleus in presence of the t(13;17). Mean values for each condition are represented above the violin plot with the p-value of the corresponding t-test. C. Comparison of SSC17 distribution along the medio-lateral axis in sperm nuclei from control (gold yellow) and t(13;17) (dark green) animals. Mean values for each condition are represented above the violin plot with the p-value of the corresponding t-test.

To go deeper in the analysis, we measured the distance of the center of each CT to the border of the nucleus. As expected, a significant difference between the repartition of each CT is observed (Fig. 6A). SSC13 is mostly localized at the periphery of the nucleus with less than 20% of the analyzed nuclei containing a SSC13 CT in the central part of the nucleus. SSC17 exhibits a more uniform distribution along the medio-lateral axis of the nucleus (Fig. 6A) and this repartition is also true in lymphocytes (Fig. S1).

We then compared the spatial localization of SSC13 in sperm from control or t(13;17) animals and we observed a significant displacement of SSC13 CT to the central region of the nucleus (Fig. 6B) with 51% of the analyzed nuclei containing a SSC13 CT in the central part of the nucleus. In contrast to SSC13, SSC17 is not significantly affected and remains uniformly distributed along the medio-lateral axis (Fig. 6C).

Our results finally show that the fusion of chromosome 13 and 17 locally affect the nuclear organization by displacing the SSC13 CT from the periphery to the central part of the sperm nucleus. This displacement is also associated to a change of the CT morphology, with a more frequent rounded morphology than in the control nuclei.

## Discussion

### Nuclear Architecture in Pig Sperm Nucleus

Pioneer studies on genome architecture in the human sperm nucleus clearly showed a chromosome hairpin-like configuration. Centromeres are located into compact chromocenters near the center of the nucleus while telomeres stay in the periphery of the nucleus, forming small clusters corresponding to interactions between ends of chromosomes [13]. Comparing telomere repartition in mammals, Solov’eva et al. (2004) [42] confirmed the clustering of telomeres in six different species (rat, mouse, bull, stallion, boar and human) but observed different repartition in sperm nucleus. Rat and mouse telomeres are localized in the basal part of the sperm nucleus while in other species, it seems dispersed over nuclei.

Our data confirm the presence of small clusters combining between 2–4 telomeres and uniformly dispersed within the pig sperm nucleus, like in bovine or human sperm cells [42]. This is consistent with other observations made in porcine lymphocytes and neutrophils where the mean number of telomeres per cluster was slightly higher (4–5 telomeres per cluster) [38], probably due to telomeres association from homologous chromosomes. Our study did not allow us to confirm this result in pig sperm cells but in human sperm, telomere dimers also result from specific interactions between the two ends of each chromosome [43]. Moreover a recent study from our group also clearly showed specific association of telomeres from the two ends of the same chromosome in porcine neutrophils [44].

An interesting point of the study is the organization of chromocenters in pig sperm cells. Surprisingly, metacentric and acrocentric centromeres do not behave similarly and specific associations were observed between both types. This observation supposes the existence of a non-random process for centromeres clustering in pig sperm cells as chromosomes clustered together depending on their centromeres sequences homology. Moreover the number of centromeres within the chromocenter is different depending of the chromosome subtype. Acrocentric centromeres cluster preferentially in one or two chromocenters while metacentric centromeres associate in smaller and numerous clusters. It is not well-known on the way such ordering is made possible but we can hypothesize that specific proteins of centromere subtypes allow this non-random centromere clustering during spermiogenesis. Thus identical centromeric sequences may be specifically recognized by protein complexes and/or specific centromeric chromatin may be differentially marked allowing subtypes clustering. Thus, specific epigenetic marks have been described in plant acrocentric chromosomes [45] but also in mammals with specific H3K9me3 marks on pericentromeric and centromeric chromatin and with the presence of non coding transcripts and histone variants (for a review see [46]). *In vivo* data also support this hypothesis. In mouse, a typical all-acrocentric model with identical centromeric sequences, a single and compact chromocenter is observed in sperm cells [43] like in human sperm where centromeres (that also shared identical alpha-satellite sequence) also clustered together [47]. Moreover, in human sperm, acrocentric centromeres seem organized within a structural element in the chromocenter [48]. Specific centromeric associations may also occur at meiosis and such phenomenon has been described in mice with Robertsonian metacentric chromosomes. Indeed, in heterozygous animals for Robertsonian metacentric chromosomes, isomorphic chromosomes cosegregate (metacentric with metacentric and acrocentrics with acrocentrics) [49]. In pig somatic cells (neutrophils and lymphocytes), the degree of clustering is different, with a similar situation for acrocentric and metacentric chromosomes (around 2 centromeres by chromocenter) but again, few associations were observed between centromeres of the two types of chromosomes [38]. Altogether these data are in favor of a specific interaction between isomorphic chromosomes in somatic and germinal cells, at least in the pig species, probably due to the differences between alpha-satellite sequences. A better molecular and structural characterization of centromere subtypes will help to understand how this process can occur.

Non-random positioning of chromosomes in pig sperm cells was previously described [15] and our results on chromosomes SSCX, SSCY, SSC13 and SSC17 confirm that they occupied specific or preferential spatial positions. However, gonosomes and autosomes behave differently. Gonosomes occupy a clear and restricted position, close to the center of the sperm nucleus while autosomes localization along the antero-posterior axis is variable and is mostly characterized by their inner or peripheral position within the nucleus. We observed that SSCY is located more apically than SSCX. This is coherent with previous observations in pig [15] and different from human where the X chromosome is more apical than the Y (62% versus 57%) [50]. Specific positioning of gonosomes in mammalian sperm has been described in different species suggesting a functional significance. However inconsistencies between published data complicate the interpretation. In human, pig and platypus sperm cells, X chromosome was previously described in the apical part of the nucleus [15], [16], [26] and its localization may be linked to specific paternal X inactivation after fertilization [16]. However additional data, including ours, do not support so much this hypothesis. First, other studies in human do not confirm this apical position for X or/and Y chromosomes [24], [25], [48] and clearly showed a median localization along the antero-posterior axis, like in marsupials [16] and our results in pig. Moreover human chromosome X homologous regions in chicken and platypus chromosomes are located in the middle part of the sperm [25]. Altogether these data support ancestral and predominant gonosomes localization in the median part of the sperm nucleus. We could hypothesize that the Y chromosome (which is mainly composed of repeated sequences) would be associated with heterochromatin epigenetic marks and the X chromosome (which should be associated with repressive epigenetic marks because of the specific paternal X inactivation, [51]) would locate in the center of the sperm nucleus as an heterochromatin sub-compartment [51], [52].

In contrast with gonosomes, preferential positioning of SSC13 and SSC17 along the antero-posterior axis is less clear, consistently with data from Foster et al. (2005) [15] and differs by their external or internal position within the nucleus. This particularity is conserved in diploid cells, like lymphocytes or other somatic cells [53].

Finally, our data strengthen previous data on non-random organization of chromosome territories in mammalian sperm and particularly highlight chromocenters specificity regarding chromosome subtypes and centromere associations.

### Effect of Chromosome Fusion on Sperm Nuclear Architecture

Our initial hypothesis was to ask whether the existence of a Robertsonian translocation can affect the sperm nuclear organization. Looking at SSC13 and SSC17, our first observation highlights a high proximity between the CTs of these two chromosomes in somatic and sperm cells (Fig. 6 and Fig. S1) that may facilitate, together with preferential centromere clustering, the fusion between these two chromosomes.

The carrier of this Robertsonian translocation presents a normal spermogram and a low rate of unbalanced spermatozoa (around 3%, [32]) allowing us to work on a system with the same genetic content in each nucleus. The only difference with control sperm cells stays in the fact that in one cell out of two, SSC13 and SSC17 are fused and will colocalize. A previous study on human sperm from infertile carriers of reciprocal translocation highlights a shift in the intranuclear localization of rearranged chromosomes together with an indirect effect on gonosome centromeres positions [29]. We did not observe that Robertsonian translocation t(13;17) affects the global nuclear architecture of the pig sperm nucleus (either looking at centromeres, telomeres or gonosomes). The effect of translocations on gonosomes topology described in Wiland et al. 2008 [29] may be due to the important rate of unbalanced gametes in sperm from the analyzed infertile patients [54], [55]. We observed a strong impact of the Robertsonian translocation on SSC13 position within the nucleus. Indeed, our data show that SSC17 positioning is globally not affected while SSC13 territory is located more internally and undergo a changed in morphology, shifting from an extended to a round shape. Curiously, it is the larger chromosome (218 Mb) that is displaced and not the smallest (70 Mb) suggesting that chromosome size does not drive chromosome position. We can hypothesize that centromeres are probably more important for CTs positioning. Thereby, in human sperm, chromosomes positioning was found to be driven by a specific linear order of centromeres physically interconnected in continuous arrays [48]. We propose that the resulting centromere from SSC13 and SSC17 fusion should behave like a SSC17 centromere and so the resulting fused chromosome would adopt a SSC17-like position.

## Supporting Information

Figure S1 **Chromosome territories positioning in lymphocytes from control and t(13;17) animals.** A. 3D-FISH in whole lymphocytes from control and t(13;17) animals. SSC13 (red) and SSC17 (green). B. Boxplot representing the relative position of SSC13 and SSC17 along the medio-lateral axis in control and t(13;17) sperm nuclei. Mean values in each condition are represented above the boxplot with the p-value of the corresponding t-test. C. Kernel density plot representing the relative position of SSC13 and SSC17 along the medio-lateral axis in control and t(13;17) sperm nuclei.
(TIF)
