# Supplementary figures and images for: Sperm Nuclear Architecture Is Locally Modified in Presence of a Robertsonian Translocation t(13;17)
Source: PLoS One. 2013 Oct 31;8(10):e78005. doi: 10.1371/journal.pone.0078005 (PMC3815027; doi:10.1371/journal.pone.0078005)

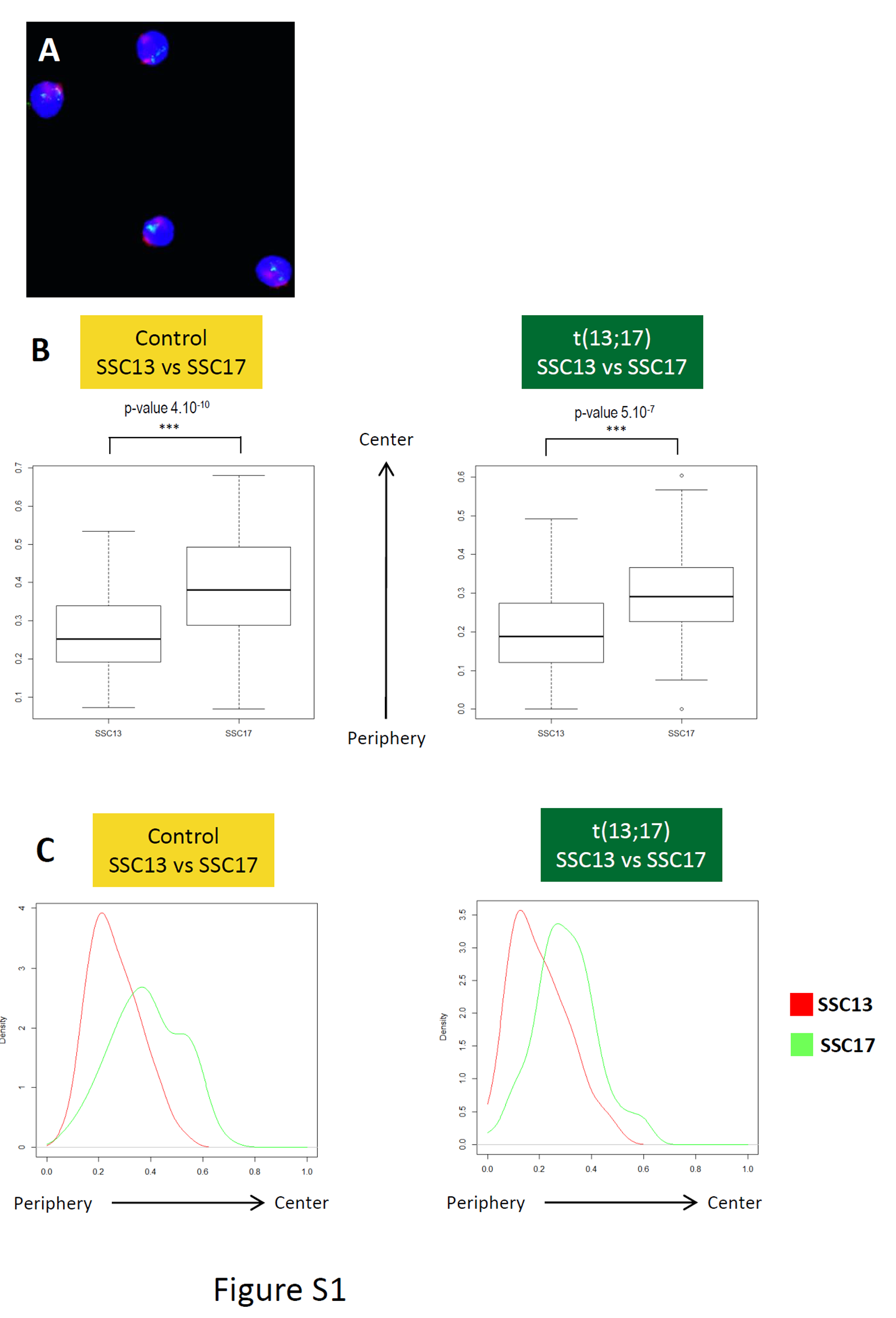

Supplement: Figure S1 — Chromosome territories positioning in lymphocytes from control and t(13;17) animals. A. 3D-FISH in whole lymphocytes from control and t(13;17) animals. SSC13 (red) and SSC17 (green). B. Boxplot representing the relative position of SSC13 and SSC17 along the medio-lateral axis in control and t(13;17) sperm nuclei. Mean values in each condition are represented above the boxplot with the p-value of the corresponding t-test. C. Kernel density plot representing the relative position of SSC13 and SSC17 along the medio-lateral axis in control and t(13;17) sperm nuclei. (TIF) [file pone.0078005.s001.tif]
